# Surface-Guided Patient Setup Versus Traditional Tattoo Markers for Radiation Therapy: Is Tattoo-Less Setup Feasible for Thorax, Abdomen and Pelvis Treatment?

**DOI:** 10.7759/cureus.28644

**Published:** 2022-08-31

**Authors:** Hui Zhao, Adam Paxton, Vikren Sarkar, Ryan G Price, Jessica Huang, Fan-Chi Frances Su, Xing Li, Prema Rassiah, Martin Szegedi, Bill Salter

**Affiliations:** 1 Radiation Oncology, University of Utah - Huntsman Cancer Institute, Salt Lake City, USA

**Keywords:** rotation, translation, radiation therapy, tattoo-less setup, surface-guided patient setup

## Abstract

Purpose: In this study, patient setup accuracy was compared between surface guidance and tattoo markers for radiation therapy treatment sites of the thorax, abdomen and pelvis.

Methods and materials: A total of 608 setups performed on 59 patients using both surface-guided and tattoo-based patient setups were analyzed. During treatment setup, patients were aligned to room lasers using their tattoos, and then the six-degree-of-freedom (6DOF) surface-guided offsets were calculated and recorded using AlignRT system. While the patient remained in the same post-tattoo setup position, target localization imaging (radiographic or ultrasound) was performed and these image-guided shifts were recorded. Finally, surface-guided vs tattoo-based offsets were compared to the final treatment position (based on radiographic or ultrasound imaging) to evaluate the accuracy of the two setup methods.

Results: The overall average offsets of tattoo-based and surface-guidance-based patient setups were comparable within 3.2 mm in three principal directions, with offsets from tattoo-based setups being slightly less. The maximum offset for tattoo setups was 2.2 cm vs. 4.3 cm for surface-guidance setups. Larger offsets (ranging from 2.0 to 4.3 cm) were observed for surface-guided setups in 14/608 setups (2.3%). For these same cases, the maximum observed tattoo-based offset was 0.7 cm. Of the cases with larger surface-guided offsets, 13/14 were for abdominal/pelvic treatment sites. Additionally, larger rotations (>3°) were recorded in 18.6% of surface-guided setups. The majority of these larger rotations were observed for abdominal and pelvic sites (~84%).

Conclusions: The small average differences observed between tattoo-based and surface-guidance-based patient setups confirm the general equivalence of the two potential methods, and the feasibility of tattoo-less patient setup. However, a significant number of larger surface-guided offsets (translational and rotational) were observed, especially in the abdominal and pelvic regions. These cases should be anticipated and contingency setup methods planned for.

## Introduction

Traditionally, patient setup for radiation therapy was accomplished by 3-5-point tattoos serving either as setup markers or as isocenter markers. The process of patient setup and positioning via tattoos is relatively simple and straightforward. Therefore, even in the image-guided radiation therapy (IGRT) era, a tattoo-based setup still remains an effective method [1]. However, there are some psychological and physiological disadvantages to applying tattoos to certain patients [2,3]. Tattoos are permanent reminders of the history of the patient's cancer diagnosis and treatments. The ink used for tattoos can diffuse under the skin causing a larger discolored skin area and, occasionally, can cause allergic reactions, skin infection, and bacteremia [4]. Patient setup using tattoos can also be time-consuming due to the need to physically move a patient who may have lain down in a slightly different posture than when tattoos were applied. Finally, there are cases where tattoos have to be positioned in an area of the skin that experiences large day-to-day variations, such as tattoos on the skin of a large belly, thereby rendering them unstable.

Surface-guided radiation therapy has been applied more regularly over the past decades and has been utilized for patient setup, target surface matching, as well as for intra-fractional motion monitoring. The matching of a patient’s surface against a reference surface on treatment day provides the possibility of automatic patient setup and positioning, which has the potential to eliminate day-to-day setup errors and inconsistency. There are multiple studies investigating surface-guided patient setup, which have proved the surface-guided patient setup can be used as a replacement for tattoo setup for a variety of treatment sites including intra-cranial, head and neck, breast/chest wall, lung, abdomen and extremities [5-21]. However, only translational comparisons have been investigated between surface-guided setup and tattoo setup in most of the non-intra-cranial studies. There is a lack of rotational evaluation of surface-guided patient setup. Leong et al. reported the observation of a maximum daily surface variation of 7.4° on the pitch, but the deviation was not corrected during patient setup [20].

It is well known that inter-fractional patient surface variations can be relatively large, in both translations and rotations, depending on treatment sites [20,22]. Therefore, evaluation of the accuracy of day-to-day surface-guided patient setup relative to tattoo setup is critical for determining the feasibility of surface-guided tattoo-less patient setup. It is also significant to evaluate the accuracy of surface-guided patient setup on treatment sites in the abdominal and pelvic region, where the large day-to-day surface variations usually happen, in both translations and rotations [20,22]. In this study, patient setup accuracy was evaluated and compared between surface guidance and tattoos for treatment sites in the region of the thorax, abdomen and pelvis.

## Materials and methods

In this study, a total of 608 setups performed on 59 patients were analyzed to compare using surface-guided and tattoo-based patient setup methods. Table 1 summarizes the treatment sites (thorax, abdomen, and pelvis) and the number of setups for each site. All patients received 3-5 point tattoos during CT simulation to serve as setup marks. Once treatment planning was finalized, each patient’s treatment plan and body contour were exported from the Eclipse treatment planning system (Version 15.5, Varian Medical Systems, Palo Alto, CA) to the AlignRT surface-guidance system (Version 5, Vision RT, London, UK) to serve as DICOM reference surface. A Region of Interest (ROI) was defined for each patient in the AlignRT system, which generally included the surface immediately around the treatment area and the side of the patient for more accurate surface vertical calculation.

The data collection workflow for this study included three steps: patient setup with tattoos, surface-guidance imaging and target localization imaging. During treatment setup, patients were aligned to room lasers using their tattoos, and then the six-degree-of-freedom (6DOF) surface-guided offsets were calculated and recorded from the AlignRT system. While the patient remained in the same post-tattoo setup position, target localization imaging (radiographic or ultrasound) was performed and the IGRT shifts were recorded. IGRT modalities in this study included kV/MV ports, kV 2D-2D match, kV-CBCT, and Clarity ultrasound system (Elekta, Stockholm, Sweden). Surface-guided vs tattoo-based setup offsets to treatment position (based on IGRT setup) were then calculated respectively and compared to evaluate the setup accuracy of the two methods. Statistical analysis of the data using a two-tailed t-test was performed on the offset differences between the two methods. This study was performed on a Varian iX Linac without a 6DOF couch, therefore, only a direct comparison of translational offsets was performed. The rotational offsets of surface guidance were evaluated separately.

**Table 1:** Number of patients and treatment fractions in each treatment site

Treatment site	# of patients	# of fractions	Percentage
Thorax	25	202	33.2%
Abdomen / Pelvis	34	406	66.8%
Total	59	608	100%

## Results

Table 2 summarizes the average difference from the final treatment position (from IGRT) to either the tattoo setup or the surface-guidance setup along the three principal directions. The results showed the overall offsets of tattoo-based and surface-guidance-based patient setups were comparable being within 3.2 mm, with tattoo-based setups showing slightly smaller offsets. The reported average difference was approximately 1 mm in the vertical direction, 3 mm in the longitudinal direction, and basically no difference in the lateral direction. Statistical analysis of the data using a two-tailed t-test showed that the differences in offsets were statistically significant in the longitudinal and lateral directions (p=0.004 and 0.00003, respectively). Table 3 shows the maximum IGRT shifts to the final treatment position from both tattoo and surface guidance along three principal directions. The overall maximum offset for the tattoo was 2.2 cm in the longitudinal direction, and the maximum offset for surface guidance was 4.3 cm in the longitudinal direction. Larger offsets (ranging from 2.0 to 4.3 cm) of surface-guided setup along longitudinal direction were observed in 14/608 cases (2.3%), and the maximum tattoo offset for these same cases was 0.7 cm. 13 of the 14 larger surface-guided setup offsets were recorded for the abdominal and pelvic treatment sites.

**Table 2:** The average difference from IGRT positioning to either tattoo or surface-guided patient setup along the three principal directions.

Treatment site	Averaged offset of tattoo			Averaged offset of surface-guidance		
	Vert (cm)	Long (cm)	Lat (cm)	Vert (cm)	Long (cm)	Lat (cm)
Thorax	0.32±0.31	0.34±0.39	0.29±0.28	0.31±0.24	0.46±0.44	0.26±0.25
Abdomen/pelvis	0.41±0.32	0.34±0.33	0.29±0.26	0.55±0.42	0.75±0.66	0.36±0.35
Overall	0.38±0.32	0.34±0.35	0.29±0.26	0.47±0.39	0.66±0.61	0.32±0.32

**Table 3:** The maximum IGRT shifts from tattoo and surface-guided patient setup along three principal directions.

Treatment site	Maximum shift from tattoo			Maximum shift from surface-guidance		
	Vert (cm)	Long (cm)	Lat (cm)	Vert (cm)	Long (cm)	Lat (cm)
Thorax	1.4	2.2	1.8	1.4	2.3	1.5
Abdomen/pelvis	1.8	1.9	1.5	2.2	4.3	3.3

Larger rotations (>3° in any yaw, roll or pitch direction) were observed in 18.6% of surface-guided setups. Table 4 shows the number of setups where rotations (yaw, roll, and pitch) fell into the range of 3°-5°, 5°-10°, and over 10°. 84% of rotations over 3° were observed for abdominal and pelvic sites, vs. 92% of rotations over 5° for abdominal and pelvic sites. Table 5 summarizes the detailed occurrences of rotations over 3° on yaw, roll and pitch for each treatment site. The reported results showed the majority of larger rotations over 3° were observed on the pitch (213/339 cases, 62.8%).

**Table 4:** Number of rotations in any direction recorded by the surface-guided setup in each range for each treatment site

Treatment site	3° – 5°	5° – 10°	Over 10°
Thorax	46	8	1
Abdomen / Pelvis	183	87	14
Total	229 (12.6%)	95 (5.2%)	15 (0.8%)

**Table 5:** Number of yaw, roll and pitch for rotations over 3° in each range for each treatment site

Treatment site	Thorax			Abdomen / Pelvis		
	yaw	roll	pitch	yaw	roll	pitch
3° – 5°	14	9	23 (50%)	54	23	106 (57.9%)
5° – 10°	1	2	5 (62.5%)	10	9	68 (78.2%)
Over 10°	0	1	0	2	1	11 (78.6%)

## Discussion

The result of an average translational difference in IGRT shifts between tattoo-based and surface-guidance-based setups in our study agreed with published data [17-19]. However, no rotational offsets were recorded and evaluated in most of the published studies. Observation of daily surface variation on pitch reached 7.4° was mentioned by Leong et al. [20], but no correction and action were discussed in the paper.

The larger surface-guided translational and rotational offsets observed in the abdomen and pelvis patients were not unexpected [20, 22]. An earlier study [22] by our group showed large day-to-day variations of relative position between target volume and patient surface in the pelvic region, with significant pelvic surface day-to-day variations due to bladder and bowel fillings and patient weight change. For the scenario of a tattoo-less setup using surface guidance, it is relatively simple to correct translational offsets. After IGRT, the translational offsets can be corrected by shifting the couch accordingly, even if the shifts are fairly large. A significant question raised here is how to deal with rotations suggested by a surface-guided setup. While a 6DOF couch can correct for some rotations, these are typically limited in the amount of rotation they can apply either due to mechanical limitations or from a patient safety perspective. In this study, 18.6% of surface-guided setups showed rotations exceeded 3°, while the IGRT showed the targets were relatively well positioned. Therefore, it is questionable whether the patient’s position needs to be corrected by the rotations suggested by surface guidance during setup. Another significant question for targets deeply seated under the skin is how much of the rotations shown by surface guidance are really due to the patient setup position variations, but not the patient's day-to-day surface variations. If the patient rotations were corrected by the amount suggested by surface guidance during setup, the consecutive IGRT would likely show the surface-guided rotation corrections were overestimated or unnecessary, and the patient would need to be shifted back towards their original position. Furthermore, it is hard to correct for rotations greater than 5°.

It should also be mentioned that Sueyoshi et al. reported another method for tattoo-less patient setup: embedded couch coordinates during treatment planning with verification of surface-guided setup [23]. This approach eliminated the treatment couch shifts before IGRT; however, it did not resolve the surface-guided setup offset issues, since the correction of patient day-to-day setup variations still relied on surface guidance. Even if the couch position was set, the patient surface-guided offsets still needed to be corrected.

The larger surface-guided setup translational and rotational offsets observed in some cases in our study show the importance of improving the workflow of surface-guidance-based patient setup. Surface-guided setup based on ROI and the limitation of field-of-view only to the surrounding treatment area might not be enough for an effective tattoo-less setup, especially in the abdominal and pelvic region. An entire body pose snapshot check may significantly facilitate tattoo-less setup, and make it possible that the limited field-of-view on large day-to-day variation patient surface ROI surrounding treatment area is not the only choice for surface-guided setup. However, most commercially available surface-guidance systems have only sets of cameras focusing on the treatment isocenter, therefore lacking the ability to check a patient’s entire body pose. The surface matching on orthopedic head-to-toe patient posture setup may provide significant information for tattoo-less setup in the abdominal and pelvic region [24]. Therefore, we believe an accurate patient posture alignment combined with applying the surface-guided translational offsets may be a promising solution for tattoo-less setup in the abdominal and pelvic region.

There were several limitations of our study, including no 6DOF couch, limited choices of characteristic skin area as an ROI in the abdominal and pelvic regions, and inevitable modest surface covering of the patient. One significant result of this study was larger rotations recorded by surface-guided setup, which was unrelated to the treatment couch. The choices of ROIs in abdominal and pelvic regions are generally limited to the skin areas which lack features for surface matching and are vulnerable to day-to-day variations. Furthermore, any modest covering used would lead to even further restrictions in the ROI choice, since the covering distorts the real patient surface.

## Conclusions

In summary, the small averaged differences in this study between the two setups confirm the general equivalence of tattoo-based and surface-guidance-based patient setups, and the feasibility of surface-guidance-based tattoo-less setup. However, a significant number of large surface-guided offsets (translational and rotational) were observed, especially in the abdominal and pelvic region, which suggest such deviations should be anticipated and planned for. The necessity of extra information (such as matching of the patient's entire body) for aiding and improving the efficiency of surface-guided tattoo-less setup may become significant.
